# Comparative Evaluation of Large Language Models for Reporting Jaw Lesions on Panoramic Radiographs

**DOI:** 10.3390/diagnostics16132064

**Published:** 2026-07-01

**Authors:** Duygu Çelik Özen, Okan Özen, Utku Tuğberk Göktürk, Hamzahan Solak, Şuayip Burak Duman

**Affiliations:** 1 Department of Oral and Maxillofacial Radiology, Faculty of Dentistry, Inonu University, 44280 Malatya, Turkey; 2 Department of Periodontology, Faculty of Dentistry, Inonu University, 44280 Malatya, Turkey; 3 Department of Diagnostic Sciences, Texas A&M College of Dentistry, Dallas, TX 75246, USA

**Keywords:** large language models, panoramic radiography, jaw lesions

## Abstract

**Background/Objectives:** The aim of this study was to assess the diagnostic capabilities of three large language model-based artificial intelligence chatbots (ChatGPT 4.0, Gemini 2.5, and Microsoft Copilot) in the radiographic evaluation of jaw lesions on panoramic images with different densities (mixed, radiolucent, and radiopaque). **Methods:** 120 panoramic radiographs showing jaw lesions with varying radiographic appearances were independently analyzed using three artificial intelligence chatbot systems. Each model was provided with the same single-round prompt and a standardized diagnostic scoring framework encompassing lesion structure, configuration, border characteristics, morphology, relationship with teeth, effects on adjacent structures, biological behavior indicators, and total diagnostic scores. Descriptive statistics were reported as mean ± standard deviation and median (minimum-maximum). Differences between LLM scores were analyzed using the Kruskal–Wallis test, followed by Bonferroni-corrected post hoc comparisons. The statistical significance level was set at *p* < 0.05. **Results:** Significant differences were observed among the LLMs across multiple diagnostic categories, including lesion structure, configuration, border characteristics, and total scores (*p* < 0.05). Gemini achieved the highest total scores in radiolucent (11.49 ± 4.97) and mixed lesions (9.01 ± 5.78), whereas ChatGPT showed slightly higher performance in radiopaque lesions (10.93 ± 2.88). Copilot demonstrated the lowest overall performance across all lesion categories. **Conclusions**: Large language model–based artificial intelligence chatbots showed variable performance in the panoramic radiographic evaluation of jaw lesions with radiolucent, radiopaque, and mixed patterns, suggesting potential utility as supportive tools. However, further validation studies are required before routine clinical implementation.

## 1. Introduction

Artificial intelligence (AI) refers to systems capable of learning from data, generating outputs, and adapting to changing conditions through training and experience [1]. The primary objective of this technology is to develop systems that can learn from data and feedback, reason, and adapt to changing environmental conditions. Large language models (LLMs) are a type of generative artificial intelligence (Gen-AI) model trained on large amounts of textual data and capable of producing human-like text based on the provided input [2,3]. ChatGPT (OpenAI), Gemini (Google), and Microsoft Copilot are among the most widely used LLM-based AI chatbots. These systems can process multimodal inputs, including text and images [4,5,6].

The accuracy of chatbots in answering clinically relevant questions in dentistry has been investigated across numerous subfields, including pediatric dentistry [7], operative dentistry and endodontics [8], orthodontics [9], periodontology [10], implantology [11], dental trauma [12], oral and maxillofacial surgery [13], oral pathology [14], and maxillofacial radiology [15]. Overall, AI has the potential to improve clinical efficiency and support diagnostic decision-making in dentistry. However, despite growing interest in LLMs, most existing studies have primarily focused on text-based performance rather than radiographic image interpretation [7,8,9,10,11,12,13,14,15]. Oral and maxillofacial radiology represents a promising field within dentistry for the use of multimodal LLM-based chatbots. While these systems provide support for text-based tasks, they can also address the growing need for decision support in the evaluation of head and neck pathologies and in report generation based on dental radiographs [16].

Radiological evaluation for diagnosis and treatment planning is a common and fundamental practice in dentistry. Panoramic radiographs enable visualization of the teeth, the mandible, a large portion of the maxilla, the hard palate, and both temporomandibular joints in a single exposure, and they serve as a useful initial screening tool for identifying pathological lesions [17,18]. Maxillofacial lesions may appear as radiolucent, mixed, or radiopaque on radiographs, depending on the characteristics and amount of the tissue they contain [19]. Accurate and clear reporting of these lesions helps clarify the diagnostic process and strengthens treatment decisions. It has been reported that reporting errors and missed findings are associated with both human factors and cognitive biases, and that discordance rates in certain radiological examinations can reach up to 30% [20].

Despite growing interest in LLMs, most previous studies have focused on text-based tasks rather than direct radiographic image interpretation. However, dental diagnosis relies heavily on radiographic imaging, particularly panoramic radiography, which remains the most widely used first-line imaging method in routine dental practice. Interpretation of panoramic radiographs is susceptible to subjective variability and reporting errors [20]. Therefore, evaluating the performance of current AI models on panoramic radiographic lesions may help clarify their potential role in radiographic interpretation and clinical decision support.

The aim of this study was to evaluate the potential contribution of these models to diagnostic processes by comparing the performance of ChatGPT, Gemini, and Microsoft Copilot in reporting radiolucent, radiopaque, and mixed jaw lesions on panoramic radiographs.

## 2. Material and Methods

The study was approved by the Scientific Research Ethics Committee of Inonu University Health Sciences (Approval No. 2026/9060; Approval Date: 24 February 2026) and was conducted in accordance with the principles of the Declaration of Helsinki (1975, revised in 2013). Administrative permission was obtained to access anonymized radiographic records. Due to the retrospective nature of the study and the use of fully anonymized data, the requirement for informed consent was waived by the Scientific Research Ethics Committee of Inonu University Health Sciences.

### 2.1. Selection of Images

An a priori power analysis was performed using G*Power software (version 3.1.9.7; Franz Faul, Universitat Kiel, Kiel, Germany). Since directly comparable studies evaluating multiple LLMs across different lesion types are limited, a medium effect size (f = 0.25) was assumed with reference to the available literature [21] on AI-based radiographic lesion assessment. With an alpha level of 0.05 and a desired statistical power of 0.90, the minimum required sample size was calculated as 78 panoramic radiographs. To improve the stability of subgroup comparisons and allow a balanced distribution across lesion types, the sample size was increased to 120 panoramic radiographs, with 40 images included for each lesion type.

For the study, panoramic radiographs were identified from the radiology archive of the Department of Radiology, Faculty of Dentistry, Inonu University. The radiographs had been obtained using a single panoramic imaging system (Planmeca ProMax 2D; Planmeca Oy, Helsinki, Finland), with image acquisition parameters of 66 kV, 8 mA, and 15.8 s. The radiographs belonged to patients aged 18 years or older with permanent dentition and contained a single radiolucent, radiopaque, or mixed (radiolucent/radiopaque) jaw lesion located in either the maxilla or the mandible. A total of 120 panoramic radiographs, with 40 from each group, were anonymized and saved in JPG format.

### 2.2. Diagnostic Criteria

Two experienced oral and maxillofacial radiologists (DCO, SBD) evaluated all panoramic radiographs by consensus and systematically characterized the lesions according to the standardized parameter set used in the study [21]. During this evaluation process, under the heading of lesion structure, radiographic density (radiolucent, radiopaque, mixed) and internal characteristics (homogeneous, heterogeneous, septated) were assessed; under the heading of configuration, loculation (unilocular, multilocular) and contour patterns (smooth, lobulated, irregular) were evaluated; under the heading of border characteristics, margin definition (well-defined, ill-defined, infiltrative) and the nature of the peripheral line (sclerotic, partially sclerotic, or absent) were recorded; under the heading of morphology, overall shape (round, oval, scalloped, irregular), symmetry (symmetric, asymmetric), and regional distribution (anterior, posterior, angle, ramus, body, symphysis) were determined; under the heading of relationship with teeth, the associated teeth (incisor, canine, premolar, molar, none), positional relationships to the tooth (pericoronal, periapical, lateral root contact), and effects on teeth (root displacement, resorption, or none) were documented; under the heading of effects on adjacent structures, neurovascular structures (mandibular canal, mental foramen, nasopalatine canal, no relationship) and adjacent spaces (maxillary sinus, nasal cavity, none) were evaluated; and under the heading of biological behavior indicators, the possible origin (odontogenic or non-odontogenic) and radiological nature (developmental, inflammatory, neoplastic) were individually defined (Table 1). Only lesions for which both reviewers reached agreement across all evaluated criteria were included in the final analysis.

### 2.3. Reporting Lesions in Panoramic Radiographs Using LLMs

For the diagnostic evaluation of lesions in panoramic images, three different LLM assistants were used: ChatGPT 4.0 (OpenAI), Gemini 2.5 (Google), and Copilot (Microsoft). All models were accessed through their publicly available browser-based interfaces and were evaluated using default settings without customized instructions or parameter modifications. Each model was operated in a separate session using the corresponding panoramic image and diagnostic table (Table 1). No lesion annotation, highlighted region of interest, or localization guidance was provided before image submission. All model evaluations were conducted in a single-turn interaction to prevent external information transfer. A new chat session was initiated for each panoramic image, and no multi-turn feedback loops were used. Thus, knowledge accumulation, transfer effects, and model memory-related biases were eliminated. The prompt provided to the models was structured to require the evaluation and reporting of the lesion in accordance with the diagnostic criteria listed in the table: “Evaluate and report the lesion in the provided panoramic radiograph according to the diagnostic criteria listed in the table.” After the prompt was defined, no additional iterations were performed, and the same prompt was used for all models.

The outputs generated by the chatbots were jointly reviewed by two experienced oral and maxillofacial radiologists (DCO, SBD) by consensus. The order of image evaluation was determined randomly, and no grouping was applied. Panoramic radiographs considered unsuitable for diagnostic evaluation due to inadequate image quality were excluded from the study. All panoramic radiographs were uploaded in their original digital format without additional preprocessing, enhancement, or image modification.

During the review, descriptive statements related to the lesions, diagnostic concepts, and key terms used for classification purposes were extracted and scored. When the key terms used by the LLMs exactly matched the lesion type defined in the reference standard, the description was considered correct and assigned a score of 1. If the LLMs correctly identified the general lesion characteristic but did not fully match the predefined reference category or subclassification, the description was considered partially correct and assigned a score of 0.5. For example, descriptions such as “well-defined” instead of “partially well-defined” were scored as partially correct. Finally, if the key term used by the LLM did not match the reference standard, if the lesion type was incorrectly identified, or if multiple key terms were used for the same lesion, with one being correct and another incorrect, the description was considered incorrect and assigned a score of 0 [21]. The chatbot outputs were independently evaluated by the radiologists according to predefined diagnostic criteria, and these evaluations were subsequently used for statistical analysis.

### 2.4. Statistical Analysis

The distribution of numerical variables was assessed using graphical methods and the Shapiro–Wilk test. Descriptive statistics were expressed as mean ± standard deviation and median (min–max). Comparisons of lesion structure, configuration, border characteristics, morphology, relationship with teeth, effects on adjacent structures, biological behavior indicators, and total scores among LLMs were performed using the Kruskal–Wallis test. Bonferroni correction was applied for pairwise comparisons. Effect sizes (ε^2^) and 95% confidence intervals were also calculated. Analyses were conducted using IBM SPSS Statistics version 26.0 (IBM Corp., Armonk, NY, USA) and Microsoft Excel 2024 (Microsoft Corporation, Redmond, WA, USA). A *p*-value < 0.05 was considered statistically significant.

## 3. Results

**Figure 1:**
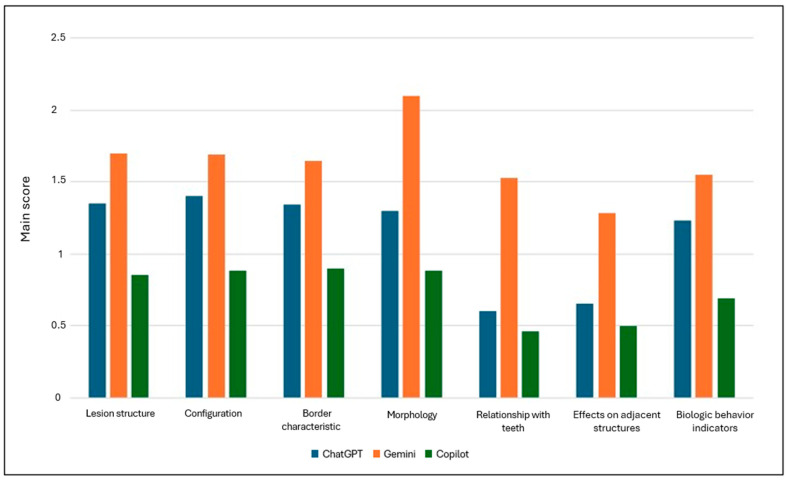
Mean scores of LLMs in reporting radiolucent lesions in panoramic radiography.

There were significant differences in the radiographic evaluation scores of the LLMs for radiolucent lesions across all evaluated parameters (*p* < 0.05). In terms of lesion structure, configuration, border characteristics, morphology, relationship with teeth, effects on adjacent structures, and biological behavior indicators, Gemini had the highest average scores. In contrast, ChatGPT performed moderately well, and Copilot had the lowest overall scores. Additionally, there was a statistically significant difference in the total scores among the groups (*p* < 0.05) (Table 2, Figure 1).

**Figure 2:**
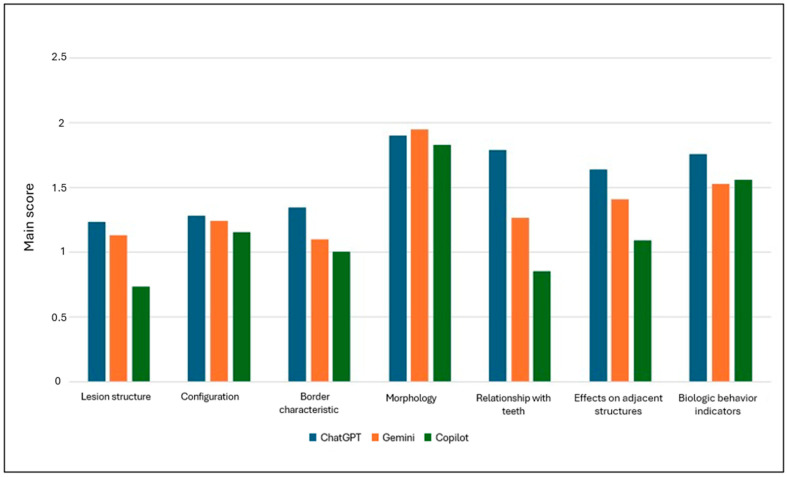
Mean scores of LLMs in reporting radiopaque lesions in panoramic radiography.

Radiographic evaluation scores of LLMs for radiopaque lesions showed significant differences in certain parameters. Statistically meaningful variations were identified among the groups in terms of lesion structure and border characteristics. Significant differences were also observed for the parameters related to the relationship with teeth and the effects on adjacent structures. In contrast, no significant differences were found between the groups regarding configuration, morphology, or indicators of biological behavior. With respect to the total score, a marked difference was detected across the groups (*p* < 0.05) (Table 3, Figure 2).

**Figure 3:**
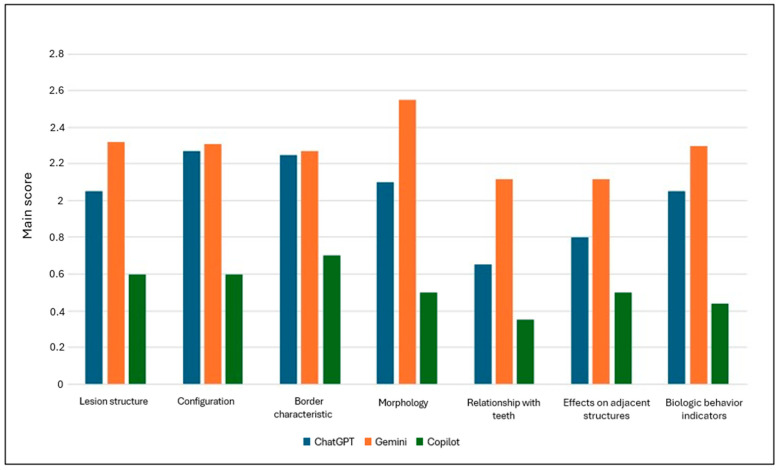
Mean scores of LLMs in reporting mixed lesions in panoramic radiography.

There were significant differences in the radiographic evaluation scores of LLMs for mixed lesions across all evaluated parameters (*p* < 0.05). Regarding lesion structure, configuration, border characteristics, morphology, relationship with teeth, effects on adjacent structures, and biological behavior indicators, statistically significant differences were found between the groups (Table 4, Figure 3).

## 4. Discussion

The performance of LLMs in lesion reporting on panoramic radiographs varied significantly depending on the lesion type and the evaluated diagnostic parameters. While all models showed limitations in image-based diagnostic tasks, distinct differences were observed between radiolucent, radiopaque, and mixed lesions. Gemini and ChatGPT generally achieved higher scores across multiple radiological criteria, whereas Copilot consistently performed worse. These performance differences may reflect variations in multimodal training strategies and visual–language integration mechanisms. However, the exact contributing factors cannot be determined from the current study design.

Model performance declined noticeably in more complex radiographic patterns, particularly in mixed lesions. These entities require the simultaneous assessment of radiolucent and radiopaque components with internal architectural features, cortical continuity, and density gradients. Such multi-component evaluations appear to present greater interpretive challenges than more uniform patterns. This may be particularly challenging for general-purpose models that were not specifically trained on dental radiographic data. While radiopaque lesions often exhibit more sharply defined density patterns, radiolucent and mixed lesions generally require broader contextual interpretation and more advanced radiological analysis, especially for parameters involving adjacent anatomical structures and indicators of biological behavior.

Previous studies have also reported that LLM-generated outputs tend to be less satisfactory when dealing with more complex pathological categories. In fact, accuracy rates for LLMs in radiology-related tasks range from 37% to 92.5%, with lower consistency observed in complex or ambiguous diagnostic scenarios [3]; such variability also reflects differences in study design, prompt strategies, and the nature of the tasks evaluated. Regarding dental imaging, the panoramic radiograph-based evaluation of radiolucent lesions has been addressed in a previous study, where ChatGPT-4 was reported to demonstrate high performance in certain categories [22]. However, it has been suggested that this apparent success may have been influenced by the use of links directing to freely accessible online images accompanied by detailed textual descriptions. In the present study, in contrast, original images uploaded directly were used, thereby reducing the risk of such bias. This methodological difference is a plausible explanation for the lower scores observed in the current study, though other factors, including lesion complexity and scoring criteria, may also have contributed.

In a study based on 100 real radiolucent jaw lesion cases, ChatGPT-4o achieved an accuracy of 66% using panoramic images alone, while the inclusion of computed tomography and histopathological information increased the accuracy to 82% [23]. Gemini, however, demonstrated lower performance under the same conditions, with only a limited improvement observed. Li et al. [24] reported that GPT-4 achieved higher accuracy than GPT-3.5 in extracting additional imaging recommendations from radiology reports, including modality, body part, time frame, and rationale, suggesting that LLMs show comparatively stronger performance in structured text extraction tasks.

Nevertheless, in a study focusing on report generation from panoramic radiograph-based evaluation, Stephan et al. [25] emphasized that although ChatGPT produced textually readable and error-free reports, it frequently omitted critical diagnostic information, a pattern also evident in the current study, particularly for parameters related to adjacent anatomical structures and biological behavior indicators. This observation is consistent with the lower performance observed in complex lesion categories in the present study. Furthermore, an evaluation of panoramic anatomical landmark recognition reported that ChatGPT-4o correctly identified only 71% of 35 anatomical structures, with high error rates observed, particularly in regions such as the inferior nasal concha. When these studies are considered collectively, it becomes evident that LLMs exhibit meaningful variation in performance depending on task structure and lesion complexity. Moreover, panoramic radiograph interpretation, which involves substantial visual anatomical variability, currently represents a domain where model reliability cannot yet be assumed.

When the subparameters used for lesion report generation were examined in this study, it was noteworthy that significant differences emerged even in fundamental radiographic criteria, such as lesion structure and border characteristics. In particular, Copilot’s low scores in categories requiring more complex interpretation, such as the effects on adjacent structures and the relationship of the lesion with teeth, suggest that this model was less capable of addressing spatial relationships and contextual anatomical features in the current evaluation setting.

On the other hand, some studies have suggested that LLM performance may improve when additional contextual information is provided. Kahalian et al. [26] reported a marked increase in diagnostic accuracy when supplementary diagnostic cues were supplied to GPT-4. Similarly, the variable scores produced by the models in more abstract parameters, such as morphology and indicators of biological behavior in the present study, further highlight the importance of structured data presentation and standardized prompt design. In addition, the large number of diagnostic subcategories and assessment parameters used in the present study may also have increased task complexity and influenced model performance. Evaluating multiple radiographic features simultaneously may be more challenging for general-purpose AI systems, particularly in the presence of panoramic radiographs containing heterogeneous lesion characteristics.

In the context of diagnosis and treatment recommendations, Uranbey et al. [27] reported that ChatGPT achieved scores comparable to clinicians in generating differential diagnoses; however, they emphasized that expert supervision remains essential due to the risk of errors. Hu et al. [28] evaluated the potential of ChatGPT to establish diagnoses based on chief complaints and radiological findings from cone-beam computed tomography (CBCT). In their study, including 102 complex oral and maxillofacial cases, an overall accuracy score of 3.7 was reported. The authors noted that performance in generating pathological diagnoses was particularly limited, especially for neoplastic and cystic diseases, which aligns with the lower scores observed for complex and mixed lesions in the present study.

For example, Silva et al. [29] reported that ChatGPT-4 and Microsoft Copilot showed notably low overall performance scores in classifying third molar positions and that hallucinations were frequently observed. In their study, total scores remained in the range of 0.22–0.28, and the models were noted to occasionally classify teeth as present even when they were absent. Peker et al. [30] evaluated the reliability of ChatGPT-4o for dental age estimation on panoramic radiographs and concluded that, although the model showed some promise, its accuracy and reproducibility were inferior to established atlas-based methods, with inconsistent outputs that would need to be addressed before clinical adoption could be considered. Conversely, Asar et al. [31] demonstrated that a customized GPT-4V model could improve accuracy in the detection of supernumerary teeth, emphasizing the importance of domain-specific fine-tuning. This finding is highly relevant to the present results, suggesting that the performance gap between models observed here may be partly attributable to differences in how well each model’s training incorporated dental imaging data. Taken together, these findings indicate that current LLM outputs require expert review before being incorporated into any clinical decision-making pathway.

Evaluations conducted in the educational field also indicate that these models demonstrate variable performance. Jeong et al. [15] reported that while ChatGPT Plus achieved high accuracy in basic knowledge questions, it lagged behind students in the interpretation of radiographic images. Helvacioğlu-Yiğit et al. [32] also noted that the response quality of different chatbots varied in questions related to dentomaxillofacial radiology, emphasizing that clinical information must always be supported by expert verification. Similarly, Morishita et al. [33] demonstrated that GPT-4V showed a low accuracy rate in image-based questions from the national dental licensing examination. These findings collectively suggest that while LLMs may assist with knowledge-based or text-structured tasks in dental education and radiology, their performance on image interpretation tasks, including those encountered in the present study, remains inconsistent and dependent on the nature of the visual input.

From a clinical perspective, the findings of this study suggest that currently available general-purpose LLMs may have potential as supportive tools in radiographic evaluation; however, their outputs should be interpreted cautiously, particularly in lesions with complex radiographic features or uncertain biological behavior.

This study has several limitations. First, the evaluation was restricted to three commercially available general-purpose LLMs, excluding domain-specific or fine-tuned systems. External validation with independent datasets was not performed, and no definitive gold-standard diagnostic test was available for all lesions. Furthermore, the assessment relied solely on panoramic radiographs without the integration of CBCT findings, clinical history, or histopathological data, which may have limited the comprehensive interpretation of complex lesions. Each image was evaluated in a single session using a one-time query strategy; consequently, intra-model consistency and repeated-trial reproducibility were not assessed. A single-round prompting strategy was preferred to standardize the evaluation conditions across all models and to reduce variability related to iterative interactions. Additionally, lesion reporting and chatbot output evaluation were performed by consensus between two experienced oral and maxillofacial radiologists. Therefore, inter-rater agreement statistics were not calculated, and this consensus approach may have introduced observer-related bias. In addition, the inherent distortion, anatomical superimposition, and two-dimensional (2D) nature of panoramic radiographs may have affected the evaluation of lesion borders and adjacent anatomical structures. These findings should therefore be interpreted with caution.

## 5. Conclusions

In conclusion, the evaluated LLMs showed variable performance in the assessment of jaw lesions on panoramic radiographs. Gemini achieved higher scores in multiple radiological criteria, whereas Copilot showed lower overall performance. Model performance declined noticeably in the evaluation of complex lesions and advanced radiographic features. Within the limitations of this study, currently available LLMs may be useful as supportive tools in dentomaxillofacial radiology. However, their outputs should be interpreted with caution and always verified by an expert clinician.

## Figures and Tables

**Table 1:** Diagnostic criteria and radiographic parameters used for the evaluation of lesions.

Main Category	Subcategory	Assessment Options
Lesion Structure	Radiographic density	Radiolucent · Radiopaque · Mixed
	Internal characteristic	Homogeneous · Heterogeneous · Septated
Configuration	Loculation	Unilocular · Multilocular
	Contour pattern	Smooth · Lobulated · Irregular
Border Characteristics	Margin definition	Well-defined · Ill-defined · Infiltrative
	Peripheral line	Sclerotic · Partially sclerotic · Absent
Morphology	Overall shape	Round · Oval · Scalloped · Irregular
	Symmetry	Symmetric · Asymmetric
	Regional distribution	Anterior · Posterior · Angle · Body · Ramus · Symphysis
Relationship With Teeth	Associated teeth	Incisor · Canine · Premolar · Molar · None
	Positional relationship to the tooth	Pericoronal · Periapical · Lateral root contact
	Effects on teeth	Root displacement · Root resorption · None
Effects on Adjacent Structures	Neurovascular structures	In contact with mandibular canal, nasopalatine canal, mental foramen · None
	Adjacent cavities/spaces	Maxillary sinus · Nasal cavity · None
Biologic Behavior Indicators	Possible origin	Odontogenic · Non-odontogenic
	Radiologic nature	Developmental · Inflammatory · Neoplastic

**Table 2:** Comparison of scores across LLMs for radiolucent lesions.

Parameter	ChatGPT	Gemini	Copilot	*p*	Effect Size ε^2^ (95% CI)
Lesion structure	1.35 ± 0.83 ^a^	1.70 ± 0.65 ^b^	0.85 ± 0.89 ^c^	<0.001	0.151 (0.051–0.301)
Configuration	1.40 ± 0.84 ^a^	1.69 ± 0.67 ^b^	0.88 ± 0.91 ^c^	<0.001	0.138 (0.042–0.286)
Border characteristics	1.34 ± 0.83 ^a^	1.65 ± 0.62 ^b^	0.90 ± 0.89 ^c^	<0.001	0.117 (0.026–0.258)
Morphology	1.30 ± 1.09 ^a^	2.10 ± 1.15 ^b^	0.88 ± 1.02 ^c^	<0.001	0.174 (0.073–0.329)
Relationship with teeth	0.60 ± 0.84 ^a^	1.53 ± 1.11 ^b^	0.46 ± 0.81 ^c^	<0.001	0.172 (0.068–0.325)
Effects on adjacent structures	0.65 ± 0.74 ^a^	1.28 ± 0.88 ^b^	0.50 ± 0.75 ^c^	<0.001	0.133 (0.039–0.281)
Biologic behavior indicators	1.23 ± 0.86 ^a^	1.55 ± 0.71 ^b^	0.69 ± 0.79 ^c^	<0.001	0.157(0.056–0.309)
Total score (max 16)	7.86 ± 4.95 ^a^	11.49 ± 4.97 ^b^	5.15 ± 5.39 ^c^	<0.001	0.241 (0.119–0.392)

Statistically significant difference according to the Kruskal–Wallis test with Bonferroni-adjusted post hoc comparisons; ε^2^ effect size with 95% confidence interval (*p*  <  0.05). Different superscript letters (a, b, and c) indicate statistically significant differences between groups, whereas groups sharing the same letter are not significantly different according to pairwise comparisons with Bonferroni correction.

**Table 3:** Comparison of scores across LLMs for radiopaque lesions.

Parameter	ChatGPT	Gemini	Copilot	*p*	Effect Size ε^2^ (95% CI)
Lesion structure	1.23 ± 0.77 ^a^	1.13 ± 0.69 ^ab^	0.73 ± 0.45 ^b^	0.002	0.089 (0.011–0.231)
Configuration	1.28 ± 0.64 ^a^	1.24 ± 0.68 ^a^	1.15 ± 0.74 ^a^	0.764	0.000 (0.000–0.071)
Border characteristics	1.34 ± 0.58 ^a^	1.10 ± 0.44 ^b^	1.00 ± 0.38 ^b^	0.004	0.078 (0.006–0.214
Morphology	1.90 ± 0.81 ^a^	1.95 ± 0.81 ^a^	1.83 ± 0.50 ^a^	0.679	0.000 (0.000–0.064
Relationship with teeth	1.79 ± 1.06 ^a^	1.26 ± 1.06 ^b^	0.85 ± 0.89 ^b^	<0.001	0.113 (0.028–0.258)
Effects on adjacent structures	1.64 ± 0.58 ^a^	1.41 ± 0.54 ^ab^	1.09 ± 0.42 ^b^	<0.001	0.173 (0.067–0.327)
Biologic behavior indicators	1.76 ± 0.51 ^a^	1.53 ± 0.72 ^a^	1.56 ± 0.63 ^a^	0.206	0.010 (0.000–0.098)
Total score (max 16)	10.93 ± 2.88 ^a^	9.61 ± 2.71 ^b^	8.20 ± 2.26 ^c^	<0.001	0.197 (0.082–0.351)

Statistically significant difference according to the Kruskal–Wallis test with Bonferroni-adjusted post hoc comparisons; ε^2^ effect size with 95% confidence interval (*p*  <  0.05). Different superscript letters (a, b, and c) indicate statistically significant differences between groups, whereas groups sharing the same letter are not significantly different according to pairwise comparisons with Bonferroni correction.

**Table 4:** Comparison of scores across LLMs for mixed lesions.

Parameter	ChatGPT	Gemini	Copilot	*p*	Effect Size ε^2^ (95% CI)
Lesion structure	1.05 ± 0.71 ^a^	1.32 ± 0.86 ^b^	0.60 ± 0.78 ^c^	<0.001	0.114 (0.024–0.273)
Configuration	1.27 ± 0.85 ^a^	1.31 ± 0.92 ^a^	0.60 ± 0.81 ^b^	<0.001	0.116 (0.036–0.265)
Border characteristics	1.25 ± 0.81 ^a^	1.27 ± 0.88 ^a^	0.70 ± 0.85 ^b^	0.005	0.075 (0.008–0.209)
Morphology	1.10 ± 1.01 ^b^	1.55 ± 1.30 ^a^	0.50 ± 0.99 ^c^	<0.001	0.129 (0.035–0.287)
Relationship with teeth	0.65 ± 0.86 ^a^	1.12 ± 0.99 ^b^	0.35 ± 0.70 ^a^	<0.001	0.102 (0.023–0.246)
Effects on adjacent structures	0.80 ± 0.85 ^a^	1.12 ± 0.94 ^b^	0.50 ± 0.82 ^a^	0.008	0.065 (0.000–0.200)
Biologic behavior indicators	1.05 ± 0.85 ^a^	1.30 ± 0.78 ^b^	0.44 ± 0.63 ^c^	<0.001	0.174 (0.079–0.334)
Total score (max 16)	7.17 ± 4.65 ^a^	9.01 ± 5.78 ^b^	3.69 ± 4.47 ^c^	<0.001	0.156 (0.047–0.301)

Statistically significant difference according to the Kruskal–Wallis test with Bonferroni-adjusted post hoc comparisons; ε^2^ effect size with 95% confidence interval (*p*  <  0.05). Different superscript letters (a, b, and c) indicate statistically significant differences between groups, whereas groups sharing the same letter are not significantly different according to pairwise comparisons with Bonferroni correction.

## Data Availability

Data that support the findings of this study are available upon reasonable request.

## Abbreviations

The following abbreviations are used in this manuscript:

AI	Artificial Intelligence
Gen-AI	Generative Artificial Intelligence
LLMs	Large Language Models
